# Physician Network Breadth and Plan Quality Ratings in Medicare Advantage

**DOI:** 10.1001/jamahealthforum.2021.1816

**Published:** 2021-07-30

**Authors:** Aditi P. Sen, Mark K. Meiselbach, Kelly E. Anderson, Brian J. Miller, Daniel Polsky

**Affiliations:** Department of Health Policy and Management, Johns Hopkins Bloomberg School of Public Health, Baltimore, Maryland; Hopkins Business of Health Initiative, Johns Hopkins University, Baltimore, Maryland; Department of Health Policy and Management, Johns Hopkins Bloomberg School of Public Health, Baltimore, Maryland; Department of Health Policy and Management, Johns Hopkins Bloomberg School of Public Health, Baltimore, Maryland; Hopkins Business of Health Initiative, Johns Hopkins University, Baltimore, Maryland; Division of Hospital Medicine, Department of Medicine, Johns Hopkins University School of Medicine, Baltimore, Maryland; Johns Hopkins Carey Business School, Baltimore, Maryland; Department of Health Policy and Management, Johns Hopkins Bloomberg School of Public Health, Baltimore, Maryland; Hopkins Business of Health Initiative, Johns Hopkins University, Baltimore, Maryland; Johns Hopkins Carey Business School, Baltimore, Maryland

## Introduction

Nearly 40% of beneficiaries elect to access their Medicare benefits through Medicare Advantage (MA), in which capitated private health insurers construct physician and hospital networks and a benefits package with a minimum of Medicare Parts A and B benefits. Most plans also integrate prescription drug coverage (Part D). In exchange for network and utilization controls, beneficiaries typically receive supplemental benefits and an annual cap on out-of-pocket expenses. Medicare Advantage insurers receive a quality bonus that is tied to a star rating of 4 or greater on a 5-star scale.

If MA insurers use networks to manage costs, enrollees may face tradeoffs between cost and quality. Narrow networks may direct enrollees to cost-effective, high-quality hospitals and physicians or limit access to necessary high-cost, high-quality care. While we know network breadth varies across MA plans^1–3^ and may be associated with plan quality,^4^ in this study, we explored the extent of narrow networks across MA, types of counties where they are common, enrollment in narrow network plans, and how networks are associated with star ratings.

## Methods

We used Vericred physician networks data, publicly available US Centers for Medicare & Medicaid Services MA plan data, and Census and Area Health Resources File data on county characteristics. We examined the 2019 physician network breadth among the most prevalent MA plan designs (health maintenance organizations and preferred provider organizations), described the percentage of enrollees in narrow network plans by state, and assessed whether network breadth was associated with star ratings, adjusting for plan and county characteristics. Star ratings were calculated as a weighted average of clinical, process, and outcome measures and ranged from 1 to 5 in 0.5 increments.

We defined network breadth as the percentage of eligible county-level physicians in network, with *narrow* defined as fewer than 25% of eligible physicians. All results were weighted by plan-county enrollment. Details of data and sample construction are in the eMethods in the Supplement. Our study followed the Strengthening the Reporting of Observational Studies in Epidemiology (STROBE) reporting guidelines. Statistical analyses were performed in Stata, version 17 (StataCorp) and R, version 3.6.1 (R Foundation for Statistical Computing). Statistical significance was set at a 95% confidence level. The Johns Hopkins University institutional review board exempted this study from review because no patient data were used and all data were publicly accessible.

## Results

Our analytic sample included 44 715 plan-counties and 18 488 434 MA enrollees (82%). The mean (SD) MA network included 41.2% (27.8%) of local physicians (Table 1). Of 44 715 plan-counties, 12 552 (28%) had narrow networks and 32 163 (72%) had non-narrow networks. Among narrow network plans, 79.8% were health maintenance organizations compared with 50.7% among non-narrow plans. More narrow networks were in large metropolitan counties (40.0%) than non-narrow networks (26.7%). The mean MA penetration and mean percentage of population older than 65 years who was self-identified as Hispanic were higher in counties with narrow networks. Overall, 31% of enrolled beneficiaries were in narrow network plans. Six states had more than 50% of beneficiaries in a narrow network plan (California, Florida, Minnesota, Maryland, Wyoming, and Kansas).

The mean (SD) star rating for narrow network plans was 4.12 (0.49) compared with 3.75 (0.4) among plans with non-narrow networks (Table 2). Among narrow networks, 51.5% were associated with plans with 4.5 or more stars compared with 9.2% among non-narrow plans. In models that adjusted for plan type and county characteristics, narrow networks were associated with 0.21 more stars than non-narrow networks. Results were significant, although smaller in magnitude, with the exclusion of Kaiser plans.

## Discussion

Narrow physician networks were positively associated with star ratings. Plans may use narrow networks to achieve a higher star rating by selectively contracting with physicians and/or actively managing the quality of physicians in their network. Potential network data inaccuracies limit this study. Further, we did not investigate characteristics of narrow networks (eg, physician specialty), hospital networks, or whether high-star, narrow network plans serve beneficiaries well. Star ratings may reflect a higher quality of care; however, the evidence is mixed.^5,6^ Future work to identify the mechanisms that contribute to the positive association between narrow networks and star ratings, and implications for beneficiaries, will be important for Medicare policy.

## Supplementary Material

Supplemental Material

## Figures and Tables

**Table 1.** Characteristics of Narrow vs Non-Narrow Networks in Medicare Advantage

	Mean (SD)			
Characteristic	All	Narrow networks^b^	Non-narrow networks	Standardized mean difference^a^

Total plan-counties, No. (%)	44 715	12 552 (28)	32 163 (72)	NA
Total enrollees, No. (%)	18 488 434	5 740 427 (31)	12 748 007 (69)	NA
Network characteristics^c^				
Network breadth	41.2 (27.8)	4.9 (7.4)	57.6(15.4)	−4.36
Plan type, %				
HMO	59.7	79.8	50.7	0.51
PPO				
Local	34.3	18.1	41.6	−0.42
Regional	6.0	2.1	7.7	−0.15
County characteristics of enrollees^c^				
County type, %				
Rural/CEAC^d^	5.2	3.9	5.7	−0.05
Metropolitan/micropolitan	64.0	56.1	67.5	−0.19
Large metropolitan	30.8	40.0	26.7	0.23
Medicare Advantage penetration in county^e^	39.6 (11.3)	41.6(11.4)	38.7(11.2)	0.26
% Age ≥65 y population in county				
Female	57.0 (2.0)	57.0(1.9)	57.1 (2.0)	−0.05
Black	8.9 (10.2)	8.8 (9.8)	9.0(10.4)	−0.02
Hispanic	8.3 (13.3)	11.2 (15.2)	7.0(12.1)	0.31
Income below the poverty line	11.4(4.3)	11.9(4.4)	11.2 (4.2)	0.16
% Population in county				
No high school degree	9.8 (4.5)	10.0(4.5)	9.7 (4.6)	0.07
≥College	22.9 (6.0)	23.6(5.8)	22.6(6.0)	0.17

Abbreviations: CEAC, counties with extreme access considerations; HMO, health maintenance organization; NA, not applicable; PPO, preferred provider organization.

a Standardized mean difference calculated as Cohen *d.* An absolute value of 0.1 or higher is often used to denote a meaningful difference between means.

b Narrow networks defined as those with fewer than 25% of county physicians in-network.

c Enrollment weighted.

d CEAC counties are those designated by the US Centers for Medicare & Medicaid Services.

e Percentage of Medicare enrollees who are receiving benefits through Medicare Advantage.

**Table 2.** Association Between Network Breadth and Star Rating in Medicare Advantage

Characteristic	All	Narrow networks	Non-narrow networks	Standardized mean difference^a^

Plan star rating^b^				
Star rating of associated plans, mean (SD)	3.87 (0.46)	4.12 (0.49)	3.75 (0.40)	0.82
Plan-counties by star rating, %				
≤3 Stars	00	4.8	9.7	−0.12
3.5 Stars	32.3	19.6	38.1	−0.33
4 Stars	35.4	21.9	41.5	−0.34
≥4.5 Stars	22.3	51.5	9.2	0.81
Additional stars associated with narrow vs non-narrow networks, mean (SD)^b^				*P* value, narrow vs non-narrow
Unadjusted		0.367 (0.102)^c^		<.001
Adjusted^d^	NA	0.214 (0.058)^c^	NA	<.001
Adjusted, Kaiser excluded^d,e^		0.147 (0.062)^c^		.02

a Standardized mean difference calculated as Cohen *d.* An absolute value of 0.1 or higher is often used to denote a meaningful difference between means.

b All results are enrollment weighted.

c Robust standard error clustered at the contract level.

d Regression-adjusted association between star rating and narrow network status estimated using ordinary least squares regression adjusting for plan type, proportion of the population 65 years or older in the county who was female; Black, Hispanic, or other (includes American Indian/Native Alaskan, Asian, Hawaiian/Other Pacific Islander, and “other” from the US Census) ethnicity; and an income below the poverty line, and proportion of the county population with high school education and college education. State fixed effects included. Regression number of observations = 42 420 plan-counties.

e Number of observations = 41 786 counties.
