# Supplementary material for: Physician Network Breadth and Plan Quality Ratings in Medicare Advantage
Source: JAMA Health Forum. Author manuscript; Available in PMC 2022 Feb 7. (PMC8796886; doi:10.1001/jamahealthforum.2021.1816)
Supplement: Supplemental Material [file NIHMS1777624-supplement-Supplemental_Material.pdf]

Supplemental Online Content

Sen AP, Meiselbach MK, Anderson KE, Miller BJ, Polsky D. Physician network breadth and plan quality ratings in Medicare Advantage. *JAMA Health Forum*. 2021;2(7):e211816. doi:10.1001/jamahealthforum.2021.1816

eMethods.

This supplemental material has been provided by the authors to give readers additional information about their work.

© 2021 Sen AP et al. *JAMA Health Forum.*

eMethods

Unit of Analysis

Our overall approach was to assess Medicare Advantage (MA) network breadth and the relationship between network breadth and star ratings at the plan-county level. County is the basis for MA service areas as defined by the Centers for Medicare and Medicaid Services (CMS), including for the sake of monitoring provider network adequacy.

MA plans of the same type (e.g., HMO) offered in the same region are grouped into *contracts* by the offering insurer/"parent organization" (e.g., Aetna). Quality ratings are determined at the contract-level. Since 2008, CMS has assigned MA contracts quality ratings on a scale of 1 to 5 stars (in 0.5-star increments); since 2012, insurers receive bonus payments in the form of an increase in their benchmark for contracts rated 4 stars or higher. Ratings are intended to improve quality of care for enrollees and facilitate enrollee selection of high-quality plans and have been shown to be linked with premiums and enrollment.

In 2021, the star rating is calculated using a weighted average of 34 Part C measures (i.e., for plans with no drug coverage) and 14 Part D measures (thus, MA plans with Part D coverage are rated on all Part C and Part D measures). The contract-level quality rating assigned to each plan in the contract. Therefore, in our analytic dataset, each MA plan has a single network and a single quality rating associated with it.

Analytic Sample

As shown in the Table below, we began with the universe of MA plans, aggregated into contracts, in 2019, which included 715 contracts in 3,230 counties (22.5 million enrollees). We defined exclusion/ inclusion criteria at the contract and county levels. We limited our analysis to HMO and PPO contracts since these types are associated with clear incentives regarding networks (e.g., incentives to stay in-network to have lower cost-sharing) (resulting N=516 contracts, 3,229 counties). We further restricted our sample to contracts offered in the 48 states plus Hawaii given very low MA penetration in Alaska (resulting N=516 contracts, 3,116 counties).

We dropped counties with no Medicare providers (resulting N=516 contracts, 2,835 counties) and contract-counties with fewer than 11 enrollees since data restrictions prevent us from knowing actual enrollment in these contract-counties (resulting N=501 contracts, 2,659 counties). Finally, we kept only contracts that linked to a network in the Vericred data (resulting N=493 contracts, 2,647 counties) and for which Vericred had non-missing data for a given network (resulting N=400 contracts, 2,643 counties). Our final sample included 20,638 contract-counties, 44,715 plan-counties, and 18,488,434 MA enrollees.

Inclusion/Exclusion Step	Number of contracts	Number of enrollees	Number of contract-counties	Number of counties	Number of states/ territories
Keep all MA contracts	715	22,493,423	375,391	3,230	56
Keep HMO or PPO contracts	516	21,535,525	354,000	3,229	56
Drop Alaska, Guam, Puerto Rico, Virgin Islands, American Samoa, Northern Mariana Islands	516	20,736,355	346,121	3,116	50

© 2021 Sen AP et al. *JAMA Health Forum.*

Drop counties with no Medicare providers (i.e., no denominator)	516	20,647,413	317,444	2,835	50
Drop contract-counties with < 11 enrollees	501	20,647,413	23,526	2,659	50
Keep contracts that link to Vericred network ID	493	20,228,700	22,065	2,647	50
Keep contracts linked to Vericred networks with > 0 linked NPIs	400	18,488,434	20,638	2,643	50

Data and Variables

Our primary data source was 2019 physician network data from Vericred, a market research firm that collects network participation data from insurers and online provider network directories (through web scraping) for a variety of insurance market segments, including employer-based plans, plans purchased through the health insurance exchanges established by the Affordable Care Act, Medicaid managed care, and Medicare Advantage.

Our primary measure was plan-county network breadth. Network breadth was calculated as the percent of eligible providers in the county in the plan's network. Eligible providers (denominator) were identified as those who (1) participated in Medicare, (2) were of a specialty type identified in MA network adequacy criteria, and (3) had over five billed medical claims in the 20% sample of Traditional Medicare carrier claims. Physicians participating in each plan's network were identified in the Vericred data and used as the numerator in our measure of network breadth. Following previous work, "narrow networks" were defined as those with under 25 percent of eligible providers participating in the network.

Additional variables were 2019 MA plan characteristics, including enrollment, quality rating, type (e.g., HMO), and "parent organization" (e.g., Humana, UnitedHealth) from the Centers for Medicare & Medicaid Services (CMS), and county-level demographics, including race and education, from the Census (obtained through the Area Health Resources File). County urban/rural designation and state-level MA penetration were obtained from CMS.

© 2021 Sen AP et al. *JAMA Health Forum.*
